# The Role of Beta HPV Types and HPV-Associated Inflammatory Processes in Cutaneous Squamous Cell Carcinoma

**DOI:** 10.1155/2020/5701639

**Published:** 2020-04-06

**Authors:** Mircea Tampa, Cristina Iulia Mitran, Madalina Irina Mitran, Ilinca Nicolae, Adrian Dumitru, Clara Matei, Loredana Manolescu, Gabriela Loredana Popa, Constantin Caruntu, Simona Roxana Georgescu

**Affiliations:** ^1^“Carol Davila” University of Medicine and Pharmacy, 37 Dionisie Lupu, 020021 Bucharest, Romania; ^2^“Victor Babes” Clinical Hospital for Infectious Diseases, 281 Mihai Bravu, 030303 Bucharest, Romania; ^3^Emergency University Hospital Bucharest, 169 Splaiul Independenței, 050098 Bucharest, Romania; ^4^Colentina Clinical Hospital, 19-21 Ștefan cel Mare, 020125 Bucharest, Romania; ^5^“Prof. N. Paulescu” National Institute of Diabetes, Nutrition and Metabolic Diseases, 22-24 Gr. Manolescu, Bucharest 011233, Romania

## Abstract

Cutaneous squamous cell carcinoma (cSCC) is a common form of skin cancer with a complex but not fully understood pathogenesis. Recent research suggests the role of beta human papillomavirus (HPV) types and HPV-associated inflammatory processes in cSCC development. Beta HPV types are components of the normal flora; however, under the influence of certain cofactors, the virus may trigger a malignant process. Dysregulation of the immune system (chronic inflammation and immunosuppression), environmental factors (ultraviolet radiation), and genetic factors are the most important cofactors involved in beta HPV-related carcinogenesis. In addition, the oncoproteins E6 and E7 of beta HPV types differ biochemically from their counterparts in the structure of alpha HPV types, resulting in different mechanisms of action in carcinogenesis. The aim of our manuscript is to present an updated point of view on the involvement of beta HPV types in cSCC pathogenesis.

## 1. Introduction

Cutaneous squamous cell carcinoma (cSCC) is the second most common nonmelanoma skin cancer (NMSC) after basal cell carcinoma (BCC), originating from the keratinocytes located in the epidermis or adnexal structures. The tumour is mainly diagnosed in middle-aged and older adults, the young individuals being less frequently affected. In some cases, cSCC exhibits an aggressive behaviour with a significant risk of metastasis [1, 2]. Its pathogenesis remains incompletely elucidated; however, several risk factors have been identified. cSCC rarely occurs on healthy skin [3]. Chronic sun exposure is one of the most important factors; sun-related skin lesions, especially actinic keratoses, are described as precursors of cSCC. Therefore, it most commonly arises in the cervical region [4]. Ultraviolet- (UV-) related inflammation participates in the transformation of normal skin into a malignant tumour. Toll-like receptors- (TLR-) 4 play an important role in the inflammatory process and subsequently in the UV-associated carcinogenesis. Thus, TLR-4 expression is higher in actinic keratoses compared to normal skin and in cSCC compared to actinic keratoses. It has been shown that the inhibition of their function reduces the harmful effects of UV on the keratinocytes [5].

Chronic exposure to radiations, arsenic, or polycyclic hydrocarbons is also associated with the risk of developing cSCC [6]. Immunosuppression is an important factor, as the prevalence of cSCC in immunocompromised individuals is much higher than in immunocompetent ones [4, 7]. There are numerous studies which attest the role of human papillomavirus (HPV) in the development of SCC on mucous membranes [8]. Recent studies have pointed out the involvement of beta HPV in the pathogenesis of cSCC. It is not clear if HPV is involved only in certain pathways of cSCC development; HPV acts as a cofactor in skin carcinogenesis, or HPV is not involved in tumour development [9].

In this review, we summarize data on the incidence of beta HPV types among patients with cSCC and discuss the main mechanisms possibly involved in beta HPV-related skin carcinogenesis emphasizing the role of chronic inflammation.

## 2. HPV Replication Cycle

HPV genome comprises eight genes which are expressed in the early (E1, E2, E4, E5, E6, and E7) and late (L1 and L2) stages of the viral cycle [10]. HPV early genes participate in viral replication and modulate early viral gene products but are also responsible for virus pathogenicity, producing cell-cycle dysregulation. HPV late genes encode for capsid proteins and are especially involved in the assembly of virions and subsequently in the transmission of the infection [11].

HPV penetrates into the epidermis through microlesions. The replication cycle of the virus depends on the differentiation process of keratinocytes. The infection starts at the basal layer of the epidermis. HPV enters the cells through endocytosis, and the cell penetration facilitated by the viral proteins L1 and L2 is mediated by various components such as heparan sulfate, proteoglycans, and annexin A2; however, the cell receptor remains unknown [10, 12, 13]. After infecting the basal cells with a small number of virions, its replication becomes independent of the cell cycle, resulting in approximately 50-100 copies per cell. The viral replication, independent of the infected cell genome, is the result of the expression of E1 and E2 genes [14]. The low rate of viral replication in basal cells prevents the immune system to act against the virus [15].

Later, the infected cells leave the basal layer and reach the upper layers of the epidermis. The viral genes are overexpressed, especially E6 and E7; and the number of copies per cell exceeds 1000. The expression of E6 and E7 genes is controlled by E2 [14]. High-risk HPV types can be integrated into the cell host genome [16] in contrast to beta HPV types, which do not have this ability and persist as episomes [10, 17]. Subsequently, L1 and L2 proteins are expressed and participate in the assembly of virions [14]. The release of virions and their survival is mediated by E4 protein [15]. The target cells of HPV differ depending on HPV type; beta HPV types infect especially cells of the hair follicle bulge [15].

## 3. Classification of HPV Types

HPV is a nonenveloped virus that belongs to the *Papillomaviridae* family [18]. HPV is a ubiquitous virus that is part of the normal skin flora of immunocompetent individuals. The virus is commonly responsible for asymptomatic infections that are resolved by the host's immune system. Although numerous types, subtypes, and variants have been described, there are many similarities between them [15].

So far, over 200 types have been described and classified into 5 genera: alpha, beta, gamma, mu, and nu. In turn, each genus comprises several species and each species includes certain types. Thus, alpha papillomaviruses include species with cutaneous tropism (e.g., alpha-4—HPV types 2, 27, and 57 associated with warts) and with tropism for mucous membranes (e.g., alpha-6—HPV types 6 and 11, responsible for genital warts or alpha-9—HPV types 16, 31, 33, 35, 52, and 58 involved in cervical cancer). In fact, the vast majority of alpha papillomaviruses exhibit mucosal tropism. The beta genus comprises 5 species with cutaneous tropism including HPV 5 and 8 also known as epidermodysplasia verruciformis (EV) types, being classically reported in patients with EV. The genera gamma, mu, and nu exhibit skin tropism and cause warts [19–22]. Thus, based on their tropism, HPV types can be divided into 2 main groups, cutaneous and mucosal HPV types [23]. They can also be categorised in terms of oncogenic risk in high-risk and low-risk HPV types [24]. High-risk types are mainly involved in the development of cervical cancer and in certain cases of head and neck SCC. The main types with high-risk are HPV 16, 18, 31, 33, 35, 39, 45, 51.52, 56, 58, 59, 68, 73, and 82, and all of them belong to alpha genus [17, 20, 25].

Recent research on skin cancer has suggested that beta HPV types may also be involved in carcinogenesis, in the emergence of NMSC [26]. The hair follicle is a natural reservoir of beta HPV types. The data obtained from the analysis of the eyebrow hair follicles reflect the infection in different areas of the body [27]. Beta HPV types are acquired soon after birth, especially through the direct contact of the child with his parents. The main risk factors for beta HPV infection are organ transplant, a positive history for sunburns and old age [28]. The first reports regarding the association of HPV infection with skin cancer date from 1920s when the first case of EV was described. Later on, HPV 5 and 8 were incriminated in the progression of verrucous lesions into cSCC, these types being identified in 90% of cSCCs diagnosed in patients with EV [29, 30]. Today, the International Agency for Research on Cancer reports HPV 5 and 8 as possible etiologic agents of cSCC in patients with EV [31]. It has been revealed that HPV prevalence and the spectrum of beta HPV types in healthy skin vary according to the geographic region [32].

## 4. Is There Enough Evidence to Confirm the Etiopathogenic Role of Beta HPV Types in cSCC?

Current studies that attempt to confirm or disprove the association between beta HPV types and cSCC use various methods such as the detection of viral DNA in the skin, in eyebrow hair follicles, or in tumour tissue as well as the detection of circulating antibodies against some viral antigens [28].

### 4.1. Detection of Beta HPV DNA

Individuals are often infected with many beta HPV types. The presence of HPV DNA may represent a latent infection but the detection of antibodies denotes an active infection in the past which elicited an immune response resulting in the synthesis of antibodies [33]. It seems that an increased viral load in eyebrow hairs is associated with an important risk of cSCC [34]. Iannacone et al. [35] analyzed the presence of beta HPV DNA in eyebrow hairs in patients with cSCC and in a control group. In both groups, the proportion of HPV-positive subjects was increased but it was higher in those with cSCC (87% versus 73%). An association between cSCC and HPV 23 as well as HPV 38 was observed [35]. The study by Hampras et al. [36] included 150 patients with cSCC and examined whether the presence of beta HPV types in eyebrow hairs increases the risk of developing another SCC, but there was an inverse association between the two conditions. They noticed that the presence of beta HPV types was associated with a lower risk of developing another SCC, which may be explained by the immunogenic effect of the virus [36].

Proby et al. [33] have shown on transplantation patients that the detection of both beta HPV DNA and antibodies against the same HPV type is associated with an increased risk of developing cSCC [33]. In the study by Harwood et al. [37], beta HPV DNA was identified in 87% of immunosuppressed patients (with renal transplantation) and in 35% of the immunocompetent subjects, with the predominance of EV HPV types. The analysis was performed on both nonsun exposed and sun-exposed normal skin, and there were no differences between groups. However, an association between the presence of EV HPV DNA and the history of NMSC was observed [37]. The meta-analysis by Wang et al. [9] has shown that HPV DNA prevalence is higher in tumour tissues compared to healthy skin in both immunosuppressed and immunocompetent patients. In tumour samples, the prevalence of HPV was higher in immunosuppressed patients compared to the immunocompetent ones [9].

To determine the role of HPV in the emergence of cSCC, Arron et al. [38] evaluated the presence of papillomavirus transcripts at tumour level. They evaluated the presence of HPV and viral load in 67 cSCC samples, and in 31 cases, whole transcriptome sequencing was performed. Viral DNA (beta HPV) was detected in 30% of SCC samples, but with no differences in viral load in healthy and tumour tissue. However, the active expression of the virus in tumour samples has not been demonstrated, suggesting that the virus does not participate in maintaining the carcinogenic process [38].

The viral load is higher in actinic keratoses than in cSCC, which supports the hypothesis that beta HPV types play a role in the initiation of the carcinogenesis but not in maintaining it [39]. Some authors discuss about the “hit and run phenomenon,” which implies that beta HPV genus is involved in the initiation of tumorigenesis but not in its progression [40]. A lower prevalence of beta HPV types was observed in tumour biopsies if striping was previously performed. This result suggests that the virus is present in the superficial layers of the skin and may not be involved in pathogenesis [41, 42]. A recent study analyzed the presence of HPV in cSCC samples and in the corresponding lymph node metastases. Beta HPV DNA was identified in 9% of primary SCC tumours and in 13% of metastases concluding that HPV does not play an important role in advanced stages of SCC [43].

### 4.2. Detection of Serum Antibodies against Beta HPV Types

Data on beta HPV seroreactivity are scarce; however, antibody detection is considered a marker of an infection with increased viral load [44]. Several studies have revealed an association between beta HPV seroreactivity and cSCC [44–46]. It should also be taken into account that HPV seroreactivity increases with age. The presence of antibodies against HPV can also be regarded as an indicator of persistent infection [44].

Serological tests have revealed an increased incidence of antibodies against L1 major capsid protein of beta HPV types in the general population. Thus, in the German population an incidence of 52% was reported, and in the Italian population of 67%. However, beta HPV DNA has been identified in over 90% of individuals. Low viral load and high keratinocyte turnover may explain the lower proportion of seroconversion [25]. The study by Karagas et al. [47] performed on a large group of 2.366 patients, consisting of 663 patients with cSCC, 898 with basal cell carcinoma, and 805 controls demonstrated by serological testing a positive association between SCC and beta HPV types, an association that has not been demonstrated in the case of BCC [47].

The meta-analysis by Bzhalava et al. [48] has revealed that beta HPV (beta-1, beta-2, and beta-3 species) is the most commonly identified in SCC lesions, results obtained from both serological tests and molecular DNA detection. Serological tests identified HPV 8 (beta-1), HPV 15, HPV 17, HPV 38 (beta-2), HPV 49, and HPV 76 (beta-3) in patients with SCC in a higher proportion than in the control group. Regarding gamma, mu, and nu genera, there were no differences compared to controls, when serological tests were used [48].

In the study by Farzan et al. [49] 1.408 patients with cSCC were evaluated for the presence of antibodies against the viral protein L1. The evaluation was performed for HPV types belonging to alpha, beta, and gamma genera. Beta HPV types, especially belonging to beta-2 species, were the most commonly detected [49]. Several studies have identified beta-2 HPV types in patients with SCC; that is why it has been suggested that beta-2 types may be considered high-risk HPV types [50]. On the other hand, Iannacone et al. [51] analyzed 173 HPV-positive patients with SCC and observed an association between beta-1 HPV types and cSCC. When serological analysis was performed according to HPV type, an association between cSCC and HPV 8 and 17 (beta genus) as well as HPV 10 (alpha genus) was observed [51]. In a smaller group of patients with cSCC (46 patients), Masini et al. [52] have also showed that HPV 8 is associated with cSCC development. The results were obtained by serological testing, as in the aforementioned studies [52]. The meta-analysis performed by Chahoud et al. [29] revealed an important association between HPV 5, 8, 17, 20, and 28 and the risk of developing cSCC [29].

However, there are studies that have shown no differences concerning HPV seropositivity between patients with cSCC and controls [16]. Plasmeijer et al. [53] included 1.311 participants and tested them for the presence of antibodies to L1 capsid protein of 21 different types of HPV belonging to the beta genus. They analyzed the link between the presence of antibodies and the development of cSCC from 1992 to 2007. A total of 150 new cases were diagnosed. There was no association between the presence of antibodies against beta HPV types and the risk of developing SCC [53].

## 5. Mechanisms of Beta HPV Types Involved in the Development of cSCC

In cervical cancer, HPV infection is persistent and the viral genome is detected in all tumour cells. After the initiation of the malignant process, E6 and E7 continue to play an essential role in the propagation of the carcinogenic process [54]. In contrast, beta HPV types are involved in the initiation of carcinogenesis but not in tumour persistence and tumour growth occurring in the absence of the viral genome [55]. E6 and E7 of both alpha and beta HPV types are involved in cell cycle alteration and inhibition of apoptosis [25]. Beta HPV oncoproteins can interfere with different signaling pathways that have been shown to be altered in cSCC. Recent studies have revealed that the mechanisms by which HPV induces the development of tumours are different between alpha HPV and beta HPV types [56, 57].

### 5.1. The Role of E6

Like its counterpart in the structure of alpha HPV types, beta HPV E6 has the ability to bind to Bak, p300, as well as to cellular histone acetyltransferase. Beta HPV E6 does not bind to PDZ domains of proteins, having a different structure compared to alpha HPV E6, resulting in a lower degree of invasiveness. PDZ containing proteins are involved in cell cycle regulation. The main difference is that beta HPV E6 protein does not have the ability to degrade p53, an ability possessed by alpha HPV types, via the ubiquitin pathway. Beta HPV types does not bind to ubiquitin ligase E6AP, which is required for the degradation of p53. However, E6 proteins of HPV 5 and 8 (beta-1), most commonly incriminated in cSCC, participate in p300 degradation, blocking its phosphorylation by AKT, which leads to a decreased ATR protein level, resulting in p53 delayed accumulation in the cell and persistence of thymine dimmers, and consequently the cell ability to defend against UVB declines [56–58]. It has also been shown that HPV E6 binding p300 may lead to an increased expression of viral genes [59]. It seems that E6 of HPV 1 and HPV 8 bind the XRCC1 protein, which is involved in DNA repair [60].

On the other hand, the interference of E6 with the Notch signaling pathway was highlighted [54]. Beta HPV E6 protein binds to the transcriptional coactivator mastermind (MAML1) resulting in the inhibition of Notch function. Notch exerts a suppressive effect on squamous epithelial cells [58], and the dysregulation of its function contributes to the malignant transformation of keratinocytes [25]. In addition, Notch signaling pathway modulates the differentiation of basal cells [15].

### 5.2. The Role of E7

E7 of beta HPV types binds retinoblastoma protein (pRb) and modulates the cell cycle by inducing a quiescent state during G0 and G1 phases [57]. The study by Caldeira et al. [61] has shown that beta HPV 38 has a similar ability to alpha HPV 16 to bind pRb and interfere with its function [61]. pRb is a negative modulator of the cell entry in S phase during cell division. In contrast, HPV 8-E7 has a much lower binding capacity, approximatively 34%, and although HPV 8-E7 fails to degrade pRb, it leads to inactivation of pRb function [62]. It has been revealed that HPV 38-E7 modulates p53 activity, acting as an inducer of a dominant-negative isoform of p73, DNp73a, which exerts an inhibitory effect on both p53 and p73 [63].

It has been observed that E7 is able to induce the proliferation of stem cells-like keratinocytes [58]. In addition, E7 expression in keratinocytes may promote a decreased expression of cell differentiation markers such as calgranulin B [25]. HPV 8-E7 increases membrane-type 1 matrix metalloproteinase (MT1-MMP) expression, a proteinase involved in cell migration, which facilitates keratinocyte migration to the dermis [58]. Studies have shown that beta HPV 38 and 49 have the ability to immortalize primary keratinocytes, a characteristic that is not found in all beta HPV types [57]. The capacity of HPV 8-E6 to transform the rodent fibroblasts lines is not identified in the case of E7. However, HPV8-E7 is able to influence the viral DNA replication [60].

In an animal model, it has been found that HPV 8-E7 induces a decreased E cadherin expression and an increased N cadherin expression, which may promote epithelial-mesenchymal transition. In addition, E7-positive keratinocytes showed increased fibronectin and integrin *α*3 chain expression, which may contribute to malignant transformation [64].

## 6. Do Beta HPV Types Need Cofactors to Induce a Carcinogenic Process?

**Figure 1:**
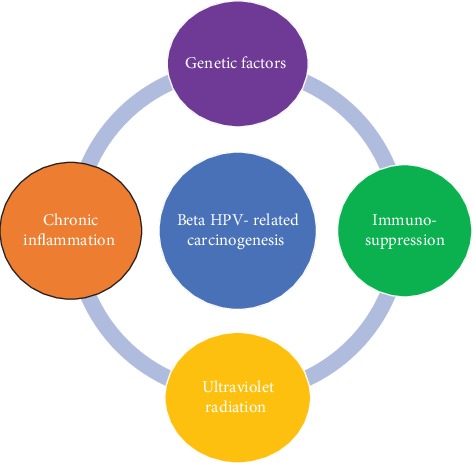
The main cofactors involved in beta HPV-related carcinogenesis.

The aforementioned studies have revealed a high incidence of beta HPV types in healthy skin. In this context, it has been speculated that beta HPV types need cofactors to induce a carcinogenic process (Figure 1). The role of ultraviolet radiation as carcinogenic factors is well known; however, the role of the immune response and chronic inflammation in carcinogenesis is a new concept incompletely elucidated.

### 6.1. Chronic Inflammation

It is well known that SCC occurs in areas characterized by chronic inflammation such as scarring or burns [65]. Inflammation is part of the body's defence mechanisms, being considered a process of the innate immunity. However, a chronic inflammatory response may become harmful to the host. Chronic inflammation contributes to tumour development and facilitates progression and metastasis. Moreover, chronic inflammation can exert a suppressive effect on the immune response [66, 67]. The cytokines released during the inflammatory response can produce genetic alterations that subsequently support the initiation of the malignant process [68, 69]. The inflammatory infiltrate and the released mediators represent important components of the tumour microenvironment that contributes to the uncontrolled cell proliferation. In the process of malignant transformation, two main pathways of inflammation are involved, the intrinsic pathway mediated by the tumour cells and the extrinsic pathway mediated by the cells of the inflammatory infiltrate [70].

The innate immune response of the host plays an important role in the evolution of HPV infection, influencing the duration and regression of the lesions. During HPV infection, keratinocytes, fibroblasts, and inflammatory cells (lymphocytes, macrophages, etc.) release an important amount of proinflammatory mediators. In some cases, HPV escape the host's defence mechanisms resulting in persistent infections. It should be noted that persistent infections are closely related to chronic inflammation, an important cofactor in carcinogenesis [71]. Tumour necrosis factor (TNF) alpha seems to be one of the most important cytokines involved in the immune response against HPV-infected cells. In addition, a recent study has revealed that HPV may influence the expression of proinflammatory genes [71]. De Andrea et al. [72] have shown that HPV 5 induces the release of numerous molecules involved in the inflammatory response such as interleukin- (IL-) 6, IL-8, intercellular adhesion molecule- (ICAM-) 1, and monocyte chemoattractant protein- (MCP-) 1, resulting in a strong inflammatory response. Thus, the host response may lead to the clearance of the infection or the infection may persist leading to the dysregulation of the immune response [72]. In line with this, Ruhland and de Villiers [73] have shown that proinflammatory cytokines, such as IL-1 alpha, IL-1 beta, TNF alpha, and IL-6 exert a stimulatory effect on HPV 20 promoter (HPV 20 belongs to EV HPV types) [73].

In the early stages of HPV infection, antiviral immunity attempts to eliminate the virus, but HPV has developed mechanisms that allow its persistence in the cells, by inhibiting acute inflammation and preventing immune recognition. In the late stages, the infected cells modify the tumour microenvironment by promoting a chronic inflammatory process. IL-6 represents an important player in this stage [74]. The activation of the IL-6/STAT3 and IL-6/C/EBP*β* pathways results in the release of chemokines and the attraction of immune cells. HPV induces CCL2 synthesis in monocytes, with a chemotactic effect on myelomonocytes. Myelomonocytes will release MMP-9, which will promote the inflammatory response [74].

It has been established that alpha HPV types are associated with chronic inflammation in cervical cancer [13]. Recent research has indicated that beta HPV types also induce inflammation. In addition, keratinocytes expressing HPV8 E2 stimulate the release of cytokines such as IL-8, with additional effect on neutrophils. In the presence of HPV 38 oncoproteins, an increased TNF alpha expression was detected. On the other hand, it was observed that HPV 38 activates NF-*κ*B in keratinocytes, which allows their survival in a cytokine-dominated microenvironment [75].

It has been shown that SCC may present a population of cells called myeloid-derived suppressor cells that are involved in the suppression of the immune response, their presence being associated with an unfavorable prognosis. Studies have revealed that HPV can stimulate these cells. Moreover, HPV seems to induce alterations in NK cells that participate in chronic latent viral infections. Certain subpopulations of NK cells exhibit immunological memory and contribute to the antitumour defence [76].

### 6.2. Immunosuppression

The prevalence of HPV infection is higher in immunosuppressed individuals than in the general population, and they have an increased risk of developing HPV-associated tumours, characterized by an aggressive behavior and a rapid growth [77]. Increased levels of HPV DNA were detected in these individuals, leading to the hypothesis that HPV infection is an important cofactor in the occurrence of SCC in these patients [78]. It seems that the presence of HPV DNA in eyebrow hair follicles and the serum antibodies to HPV double the risk of developing an SCC in transplantation patients [79]. The immunosuppression plays an important role in the persistence of HPV infection; a positive association was observed between the persistence of the virus and the low number of CD4 lymphocytes [80]. The transplantation patients have a 65- to 250-fold increased risk of developing an SCC [78]. About 40% of those who undergo organ transplantation develop NMSC within 15 years after transplantation, the majority of tumours being SCCs. Among these patients, the rate of HPV detection in cSCC samples is very high, ranging from 70 to 90% [81]. Most HPV types detected in organ transplant recipients belong to beta genus [82]. However, Shamanin et al. [83] have shown that HPV types detected in NMSC samples collected from patients with renal transplantation after the initiation of therapy are those types that are found in the genital area [83].

Clinical and histopathological analysis showed that in the case of transplantation patients most of the cSCCs arise on common warts and are located especially on the sun-exposed areas [55]. The immunosuppression and exposure to ultraviolet radiation are two factors with synergistic activity for the development of skin cancers [78]. A recent study has shown that impairing the NF-*κ*B pathway is one of the mechanisms by which HPV participates in carcinogenesis in immunosuppressed patients [84]. In the case of immunocompromised patients, differences of the tumour infiltrate were observed compared with those with a competent immune system. An increasing number of regulatory T cells was observed, which correlates with a poorer prognosis. Moreover, in immunocompetent patients, in cSCC samples, the number of Th1 and Th2 cells is similar, whereas in the immunosuppressed ones, there is a predominance of Th2 cells [76].

### 6.3. Ultraviolet Radiation

The study by Weissenborn et al. [85] analysed skin swabs collected from the forehead (79%) and the dorsal face of the hand (81%) and showed that the samples were positive for HPV (beta genus) in a higher proportion compared to those collected from the buttocks (64%), which may suggest the role of the sun in beta HPV infection [85].

Exposure to UV produces changes in the function of keratinocytes and immune cells that will release proinflammatory cytokines and promote processes such as cell proliferation and angiogenesis [76]. It seems that beta HPV types increase UV-induced inflammation and prolong cell life, being involved in the initiation of a malignant process. It can be considered that the induction of inflammation is a mechanism of adaptation of beta HPV types to a UV-activated microenvironment [75]. The link between chronic inflammation, UV radiation, and HPV is also supported by a recent study which has shown that naproxen acts on UV-related BCC and SCC, reducing the number and size of tumours. Decreased levels of cyclooxygenase- (COX-) 2, NF-*κ*B. and inducible nitric oxide synthase (iNOS) were identified [86].

However, Akgul et al. [87] demonstrated that keratinocytes infected with HPV 5 and 8 exhibit a low IL-8 expression after UVB irradiation, resulting in a poor response against UV damage which allows the accumulation of mutated cells [87].

Tomlins and Storey [88] have shown that E6 oncoprotein can inhibit UV-induced apoptosis and alter DNA repair systems in non-HPV expressing cell lines and primary human keratinocytes. E6 acts by increasing the expression of osteoprotegerin and IL-6. Furthermore, they observed the overexpression of IL-6 in HPV-positive tumours compared to HPV-negative tumours [88]. Strujik et al. [89] used primary human keratinocytes expressing E6 and E7 of a beta HPV group including HPV 5, HPV 8, HPV 15, HPV 20, HPV 24, and HPV 38 and subsequently exposed them to UVB. They observed that E6 of HPV 8 and HPV 20 inhibited UVB-induced cell apoptosis by reducing the activity of Bax protein, promoting the survival of mutated cells that might initiate the development of a malignant tumour [89].

However, Guerrini et al. [90] using HaCat keratinocytes expressing E6 and E7 have shown that E6 and E7 of a group of beta papillomaviruses including HPV 5, HPV 8, HPV 14, HPV 24, HPV 36, HPV 38, and HPV 49 do not alter UVB-induced apoptosis. They explain the results by taking into consideration that E6 and E7 can modulate apoptosis depending on the viral type and genetic factors [90]. Another mechanism by which viral oncoproteins are likely to inhibit UVB-induced cell apoptosis is related to Bak degradation. Underbrink et al. [91] demonstrated that E6 of HPV 6 and HPV 11 (alpha papillomaviruses) exert their antiapoptotic effect by degrading Bak through the proteasomal pathway [91]. Moreover, it has been demonstrated that E6 of HPV 5, -8, -20, -22, -38, -76, -92, and -96 (beta papillomaviruses) have the ability to degrade Bak, but only after the exposure to UVB [59, 91].

### 6.4. Genetic Factors

It has been postulated that certain genetic variations may increase the susceptibility to develop HPV-mediated skin cancers. It seems that genetic variations of p53 may act as a risk factor [30].

The association between beta HPV infection and cSCC is proven in the case of EV, these patients having an increased susceptibility to the infection with HPV [92]. EV is a rare autosomal recessive skin disorder. The pathogenesis of the disease involves a mutation of the EVER1 (transmembrane channel-like (TMC) 6) and EVER2 (TMC 9) genes, members of the TMC gene family, which encode for transmembrane channel-like proteins [93]. It seems that TMC6 and TMC8 are involved in intracellular zinc homeostasis, and changes of cellular zinc balance can dysregulate the life cycle of HPV [94]. Intracellular zinc homeostasis modulates HPV replication; zinc being involved in various signalling pathways [95]. In these patients, the tumours are located on the sun-exposed areas and the risk of developing a cSCC is high [93]. Up to 90% of cSCCs diagnosed in EV patients are positive for HPV 5 and 8 [94].

The mechanisms that are involved in persistent HPV 8 infections and participate in the development of neoplasms are not completely elucidated. Recent research has focused on the role of chronic inflammation. Starting from the idea that S100A8 and S100A9 proteins promote chronic inflammation and carcinogenesis in skin neoplasms, Podgorska et al. [96] have studied the role of these proteins in EV pathogenesis and detected their overexpression, especially in the upper layer of the epidermis and in the cutaneous affected areas. S100A8 and S100A9 have chemoattractant role on granulocytes that have been identified in a large number of EV lesions. It has been observed that HPV E2 is involved in the expression of these proteins. The formation of a microenvironment dominated by chronic inflammation is one of the mechanisms involved in carcinogenesis in EV patients [96].

## 7. Conclusions

The presence of beta HPV DNA in cSCC samples and the detection of antibodies against HPV in patients with cSCC are arguments in favor of the participation of beta HPV types in cSCC pathogenesis. Beta HPV infection seems to play an important role in initiating carcinogenesis, but not in tumour progression. This hypothesis is supported by the identification of an increased viral load in premalignant lesions such as actinic keratoses compared to cSCC. However, the high incidence of beta HPV types in healthy skin denotes that some cofactors contribute to its pathogenesis. We consider that chronic inflammation is a key player in beta HPV-related carcinogenesis; therefore, a deeper research is needed to understand how the inflammatory pathways are involved in the malignant process in order to counteract their activation. The data available in the literature on this topic suggest that beta HPV types should not be neglected in the onset of cSCC.
